# Molecular characterization and Functional Analysis of the PilQ_380-706_: a Novel Secretin Domain in *Pseudomonas aeruginosa*

**Published:** 2018

**Authors:** Sobhan Faezi, Iraj Nikokar, Ali Elmi, Yusuf Ghasemi, Mojtaba Farahbakhsh, Alireza Salimi Chirani, Mehdi Mahdavi

**Affiliations:** 1. Medical Biotechnology Research Center, Faculty of Paramedicine, Guilan University of Medical Sciences, Rasht, Iran; 2. Department of Mycobacteriology and Pulmonary Research, Pasteur Institute of Iran, Tehran, Iran; 3. Mycobacteriology Research Center (MRC), Tehran, Iran; 4. Laboratory of Microbiology and Immunology of Infectious Diseases, Faculty of Paramedicine, Guilan University of Medical Sciences, Rasht, Iran; 5. Department of Medical Microbiology, Faculty of Medicine, Shahid Beheshty University of Medical Science, Tehran, Iran; 6. Department of Immunology, Pasteur Institute of Iran, Tehran, Iran

**Keywords:** *Pseudomonas aeruginosa*, Proteins, Secretin

## Abstract

**Background:**

Type 4 pili (T4P) is an important virulence factor of *Pseudomonas aeruginosa (P. aeruginosa)*. T4P pass the outer membrane through a large oligomeric channel made of a single PilQ protein that is most highly conserved at their C-termini. To develop a functional vaccine that can be used in clinical application, the secretin domain of the PilQ (PilQ_380-706_) was produced as a recombinant protein.

**Methods:**

A 981 *bp* fragment of C-terminal of the *pilQ* secretin (*pilQ*_1138-2118_) from was designed into the prokaryotic expression vector pET28a. The presence of the *pilQ*_1138-2118_ gene in the recombinant construct (pET28a/*pilQ*) was assessed by double digestion and PCR. After transformation, expression of the recombinant PilQ was induced by addition of IPTG. The expressed recombinant protein was purified by a modified method using a HisTrap affinity column and finally confirmed by SDS-PAGE. The functional activities of the produced PilQ_380-706_ confirmed by Western blot analysis and twitching inhibition assay.

**Results:**

The PCR and enzymatic digestion results showed the presence of the *pilQ*_1138-2118_ gene in the construct. The protein electrophoresis showed that the molecular weight of the recombinant PilQ_380-706_ is approximately 37 *kDa*. The Western blot analysis confirmed the specificity of specific IgG against the PilQ_380-706_ protein. The PilQ_380-706_ protein showed high biological activity in all of these standard assays.

**Conclusion:**

Since, the PilQ_380-706_ protein plays an important role in the biogenesis of pili; and thus, the primary establishment of *P. aeruginosa*; it seems that it can be used as a candidate vaccine or an adjuvant in the future studies.

## Introduction

*Pseudomonas aeruginosa* (*P*. *aeruginosa*) as an opportunistic human pathogen has a remarkable capacity to cause disease in susceptible hosts. It is the major colonizing microbial pathogen for Cystic Fibrosis (CF) patients ^1^ and a common infectious agent in nosocomial infections, such as patients with a severe burn, cancer, transplantation, AIDS, and other immunocompromising conditions. Despite improvements in antibiotic therapy, *P. aeruginosa* shows inherent and acquired resistance to many antimicrobial agents ^2^. The pathogenesis of *P. aeruginosa* infections is multifactorial and is being affected by a complex of virulence factors; hence, it has made vaccine development difficult. Bacterial attachment is an initial and a critical step for the establishment of infection that involves bacterial adhesins and host receptors. One of the most important adhesins in *P. aeruginosa* is pili ^3^.

Type IV pili (T4P) are the most common type of bacterial pili and are thin, long, flexible, and retractable protein filaments. T4P are polarly localized, filamentous surface appendages present at the cell surface of a broad range of pathogenic and environmental bacterial species ^4^. This adhesive cell surface structure is the prominent virulence factor that essential for initiation of infection by mediating attachment to host cells, where non-piliated strains were reported to exhibit a 90% decrease in their ability to bind human alveolar cells ^5^, and also mutant strains that are unable to produce T4P are attenuated in virulence ^6,7^. Furthermore, another study revealed that piliated strains caused 28%–96% more cases of *P. aeruginosa* pneumonia as compared to non-piliated strains in a mouse model of infection ^8^. T4P play an important role in many processes including bacterial locomotion known as twitching motility, aggregation, infection by pilus-specific bacteriophage, DNA uptake, attachment to biotic and abiotic surfaces, host cell invasion and biofilm maturation ^9^. The pilus fiber is composed of hundred copies of PilA (or pilin, the major structural subunit) that are encoded by an operon that positively control by the *algR* regulator ^10^. The pilin monomer can be divided into three domains: a highly conserved hydrophobic N-terminal α-helix region; a hypervariable central region; and a semi-conserved C-terminal region containing β-strands. The C-terminal Receptor Binding Domain (RBD) of *P. aeruginosa* pilin is a suitable candidate for a peptide vaccine ^11^. The RBD contains a disulphide-bonded loop (DSL) that structurally is highly conserved among T4P of all species of *P. aeruginosa*, although the size of the DSL (from 12–31 amino acids) and its sequence is varied among pilin alleles.

The monoclonal antibody studies revealed that the C-terminal DSL of the pilin subunit mediates attachment to epithelium receptors, this finding suggests that PilA itself acts as both a major structural subunit and an adhesion ^7,12^. Finally, Type IV pili has a common receptor among all strains of *P. aeruginosa*; however, the sequence diversity presents a considerable obstacle to the development of a protective RBD-based vaccine targeting the T4P ^11^.

Pili are rapidly extended and retracted *via* a most powerful molecular machine that organized with four subcomplexes: the cytoplasmic motor subcomplex (consisting of PilBTUCD), the inner membrane alignment subcomplex (PilMNOP), the outer membrane secretin pore subcomplex (PilQ and PilF), and the pilus itself (or PilA) ^13^. There are significant structural and functional similarities between this pilus assembly apparatus and type II secretion system ^14^. T4P passes the outer membrane through a large oligomeric channel and makes a single protein. The PilQ (77 *kDa*; ORF PA5040) that encoded by the highly conserved *pilMNOPQ* operon ^15^ is a member of the so-called “secretin” family required for configuration of the outer membrane pore through which the pilus is extruded ^9^. The PilQ protein that is essential for T4P biogenesis consists of five conserved domains; Secretin_N (380–449), Secretin (549-705), STN (306-354), HofQ (1-707) and AMIN (63-123). The secretin domains (Secretin_N and Secretin) of the PilQ are more highly conserved at their C-termini. This region facilitates the passage of folded proteins, filamentous phage particles, DNA, and other macromolecules across the outer membrane ^16^. The secretin domain of PilQ (that’s mean PilQ_380-706_) was chosen as a new antigen and designed into expression vector pET28a.

In the present study, we designed a chimeric plasmid contains the *pilQ*_1138-2118_ gene, which codes the immunologic domains of PilQ secretin (the C-terminal domain of the PilQ). To the best of our knowledge, for the first time, we report the purification and characterization of a novel recombinant PilQ (r-PilQ_380-706_) from *P. aeruginosa*. Furthermore, our data suggest that the protein has biological activities in both *in vivo* and *in vitro* conditions.

## Materials and Methods

### Bacterial strains, plasmids, and media

*Escherichia coli* (*E. coli*) strains Top10F and BL21 (DE3) were used as preservation and expression hosts. The *P. aeruginosa* laboratory strain PAO1 (that kindly provided by Dr. Abdi from Department of Microbiology, Faculty of Biological Sciences, Alzahra University, Tehran, Iran) were performed. The recombinant plasmid pET28a/*pilQ*_1138-2118_ synthesized by Biomatik Corporation (Cambridge, Ont., Canada). All enzymes for DNA manipulations were obtained from NEB (USA). The Ni^2+^-NTA agarose was purchased from Qiagen (USA). The HRP-conjugated goat anti-rabbit IgG and Protein A/G agarose were obtained from ThermoFisher (formerly Invitrogen, USA). The strains were cultured in LB broth or on agar (HIMEDIA, India) at 37°*C* with or without 30 *μg kanamycin/ml* (Bioscience, Canada).

### Construction of the expression vector

The *pilQ*_1138-2118_ gene was inserted into the *E. coli* expression vector pET28a, in frame with a T7 promoter, kanamycin-resistant gene and the C and N-terminal six-His-tagged sequences. The gene containing BamHI and HindIII sites at the 5′ and 3′ ends, respectively. In the designation of the construct, we have inserted a start codon ATG immediately after the BamHI site (ggatccATG) of the pET28a vector, resulting in the correct framing of the gene of the insert. After transformation of the recombinant vector (pET28a/*pilQ*_1138-2118_) into *E. coli* Top10F competent cells, transformants were screened on LB plates supplemented with 30 *μg kanamycin/ml*. The recombinant vector was extracted from *E. coli* Top10F using plasmid extraction kit (Bioneer, Korea) according to manufacture instruction.

### Confirmation of the recombinant vector

The pET28a/*pilQ*_1138-2118_ vector was verified by polymerase chain reaction (PCR) and restriction enzyme digestion. The vector was treated with the restriction endonucleases BamHI and HindIII (Jena Bioscience Kit, Germany) according to manufacture instruction. The specific primers were designed for the *pilQ*_1138-2118_ sequence of the *P. aeruginosa* PAO1 strain from NCBI (Gene ID: 880962). The gene was amplified from pET28a/*pilQ*_1138-2118_ vector using the following specific primers: Forward: 5′-CCA GGT GAA CTA CGC CAA GG-3′ and Reverse: 5′-CGG TGT CAG GAA AAC CAG AAG T-3′, which incorporated by *BamHI* and *Hind*III restriction sites, respectively. Amplifications were performed in 25 *μl* volumes containing 50 *pM* of each primers, 1X PCR master mix, and 25 *ng* of the vector. The PCR was performed in a master gradient thermocycler (Bio-Rad, USA). Template DNA was denatured at 94°*C* for 4 *min* prior to the start of PCR cycling, which consisted of denaturation at 94°*C* for 46 *s*, primer annealing at 61°*C* for 1 *min*, extension at 72°*C* for 1 *min*, repeating for 30 cycles followed by a final extension at 72°*C* for 10 *min*. The digested fragments and PCR product were separated by 1.2% (*w/v*) agarose gel electrophoresis.

### Expression and isolation of inclusion bodies

In order to overexpress the protein, the recombinant construct pET28a*/pilQ*_1138-2118_ was transformed into BL21 (DE3) and platted on LB agar containing kanamycin (30 *μg/ml*). To optimize the induction conditions, the colonies carrying pET28a/*pilQ*_1138-2118_ were grown in 5 *ml* of LB medium supplemented with kanamycin at 22°*C*. At OD_600_ *nm* of 0.8, expression of the r-PilQ_380-706_ was induced by addition of IPTG (BIOSYNTH, Switzerland) to a final concentration of 1 *mM*. After 0, 1, 2, 3, 4 and 5 *hr* of induction, cells were harvested and the induced level of r-PilQ_380-706_ was determined by 12% SDS-PAGE electrophoresis.

For isolation of inclusion bodies, an overnight culture of *E. coli* BL21 (DE3) cells harboring pET28a/*pilQ*_1138-2118_ was diluted 100-fold in LB medium (1 liter) containing kanamycin and incubated at 22°*C* with shaking. When the OD_600_ of the culture reached 0.8, the promoter of the recombinant vector was induced by the addition of IPTG to the final concentration of 1 *mM*. After 4 *hr*, the induced cells were harvested by centrifugation at 8500×*g* for 10 *min* at 4°*C* and suspended in lysis buffer [20 *mM* sodium phosphate (pH=7.5), 10 *mM* EDTA, 1% (*v/v*) Triton X-100] to remove the contaminant proteins. Following freezing and thawing, the lysozyme (100 *μg/ml*) was added to lyse the cell wall and incubated for 30 *min* in room temperature. The sonication was carried out on ice in the presence of PMSF (1 *mM*) as a protease inhibitor. To chelate the EDTA and remove DNA, 10 *mM* MgSO4 and DNase (0.01 *mg/ml*) was added, respectively and followed to incubate on ice for 20 *min*. After centrifugation, the pellet was thoroughly washed with the same buffer without EDTA and resuspended again in the buffer without EDTA and Triton-X100. The Inclusion Bodies (IBs) were harvested by centrifugation and stored at 4°*C*.

### Solubilization, refolding and purification of r-PilQ_380-706_

The IBs were solubilized with Guanidinium Lysis Buffer [20 *mM* sodium phosphate, 500 *mM* NaCl, 6 *M* guanidine hydrochloride, pH=7.4]. The solubilized proteins were purified using Ni ^2+^-NTA agarose (Qiagen, USA) according to the manufacturer’s instructions with modifications. Purifications were performed under denaturing and renaturing conditions (hybrid conditions). Briefly, after applying the sample to the column and washing with denaturing binding buffer [20 *mM* sodium phosphate, 500 *mM* NaCl, 8 *M* urea, pH=7.8], a linear gradient of urea from 7 *M* to 0 *M* of refolding buffer [20 *mM* sodium phosphate, 500 *mM* NaCl, 5% (*v/v*) glycerol, pH=6.0] was used at flow rate of 0.6 *ml/min*. The contaminant proteins were washed using native wash buffer [20 *mM* sodium phosphate, 500 *mM* NaCl, 20 *mM* imidazole, pH=8.0]. Finally, bound proteins were eluted in native elution buffer [20 *mM* sodium phosphate, 500 *mM* NaCl, 250–500 *mM* imidazole, pH=8.0]. The purified r-PilQ_380-706_ was dialyzed against phosphate buffered saline (PBS, pH=7.4) for imidazole removal and analyzed by 12% (*w/v*) SDS-PAGE followed by Coomassie brilliant blue G-250 staining. The protein concentration was quantitatively measured by using a NanoDrop 2000c spectrophotometer (Thermo Scientific, USA) and Bradford protein assay using standard albumin (Sigma, USA).

### Preparation and purification of anti r-PilQ_380-706_ IgG

To determine the immunogenic nature of purified r-PilQ_380-706_, the female New Zealand white rabbits (Razi Vaccine and Serum Research Institute, Karaj, Iran) were immunized with 400 *μg* of the r-PilQ_380-706_ protein administered subcutaneously and boosted twice with 200 *μg* with 2 weeks intervals. The rabbits were anesthetized intramuscularly with an injection of a mixture of xylazine (10 *mg/kg*) and ketamine (50 *mg/kg*). The rabbits were bled prior to immunization and 2 weeks after the last immunization. Sera were collected from the retracted clot, clarified by centrifugation (2500×*g*) and then aliquoted and stored at −20°*C*. The rich fractions pooled and the specific IgGs (except IgG3) purified by using protein A/G agarose (Invitrogen, USA) according to the manufacturer’s instructions and analyzed by SDS-PAGE. Protein concentration was quantitatively determined using Nano-Drop (2000c spectrophotometer, Thermo Scientific, USA) and Bradford protein assay. Anti r-PilQ_380-706_ IgG and non-immune IgG were aliquoted at a concentration of 1–2 *mg/ml* and finally stored at −20°*C* until use.

### SDS-PAGE electrophoresis and Immunoblot analysis

The bacterial pellets and purified protein were separated by SDS-PAGE. The samples were directly resuspended at a 2:1 ratio with 3× SDS-PAGE sample buffer. in an appropriate volume of sample buffer. The discontinuous gel consisted of a 5% stacking gel and a 12% resolving gel which was run on a vertical electrophoresis unit (Mini PPROTEAN 3 cell, Bio-Rad). To determine the functional activity, the purified r-PilQ_380-706_ protein was electrophoresed, and then transferred onto a PVDF membrane (Hi-bond Amersham Biosciences, USA) by a Bio-Rad apparatus at 25 *V* for over-night. The membrane was blocked with 5% non-fat skim milk in TBST buffer (Tris buffer saline contain 0.1% Tween-20) for 2 *hr* at RT. After incubated with rabbit anti r-PilQ_380-706_ IgG (1:5000 diluted in blocking buffer) for 1 *hr* at RT, the membrane was incubated with HRP-conjugated goat anti-rabbit IgG for 1 *hr* at RT. The membrane was then washed 5 times with TBST for 5 *min* each. Finally, it was developed by adding 3, 3′-diaminobenzidine (DAB) solution (Sigma, USA) allowing it to incubate until bands were seen. The reaction was stopped by rising the membrane with water.

### Twitching inhibition assay

To verify the functionality of the r-PilQ_380-706_-specific polyclonal IgG, the twitching inhibition assay was carried out by Castric *et al* ^17^ as follows. Different concentrations (0.1, 0.2 and 0.3 *μg*) of specific rabbit anti r-PilQ_380-706_ IgG (filter-sterilized) were added to LB broth (containing 1% (*w/v*) agar), which was poured into a 15×90 *mm* plastic Petri dish. After solidification, the plate was dried for 6 *hr* at room temperature. A single colony of the *P. aeruginosa* PAO1 strain to be tested was stab-inoculated with a toothpick to the bottom of the plates. After omitting an 18 *hr* incubation at 37°*C*, the diameter zone of growth of different strains obtained at the interstitial surface of the agar and the plate was measured. For each assay, triplicate plates were examined.

### Statistical analysis

The data were analyzed using one-way analysis of variance (ANOVA) and Student’s *t*-test (StatView). Statistical analysis was performed using the software GraphPad Prism version 6.0 for Windows, (GraphPad Software, San Diego, CA, USA). All data of this study are expressed as mean±SD. The p-values less than 0.05 was considered to be statistically significant.

## Results

### Confirmation of the pET28a/pilQ_1138-2118_ construct

**Figure 1.**
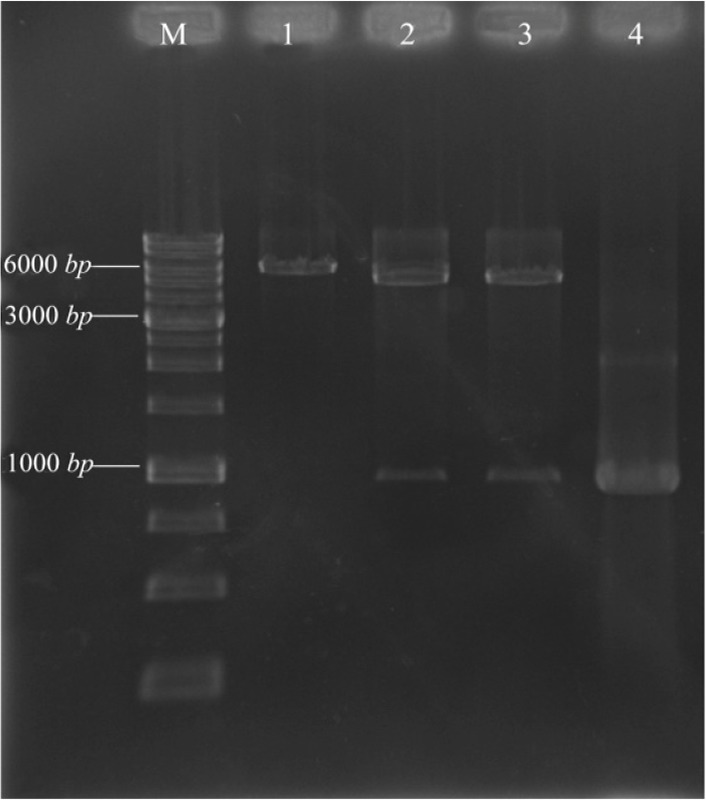
Agarose gel electrophoresis analysis of recombinant pET28a/*pilQ*_1138-2118_ with restriction enzyme digestion. Lane M; DNA marker (1 *kb*), Lane 1; mono-digestion of the pET28a/*pilQ*_1138-2118_ vector with BamHI. One expected fragment was observed on the gel (∼6350 *bp* band). Lane 2 and 3; BamHI/HindIII double digested the recombinant vector with BamHI and HindIII buffer, respectively. Two expected fragments from double digestion were observed on the gel (∼5369 and 981 *bp* bands). Lane 4; the optimized PCR product of the *pilQ*_1138-2118_ gene (∼ 961 *bp* band).

The coding sequence of the secretin domain of *pilQ* (*pilQ*_1138-2118_) was constructed in the pET28a expression vector. Transformants were characterized by enzymatic digestion. The recombinant plasmid, pET28a/*pilQ*_1138-2118_, was extracted and its orientation confirmed by digestion with two restriction enzymes that mentioned above. The target fragments with the expected sizes are shown in figure 1. DNA gel electrophoresis of the PCR product resulted in single 981 *bp* band, which confirmed amplification of *pilQ*_1138-2118_ gene (Figure 1). Sequence analysis of recombinant pET26b/*pilQ*_1138-2118_ confirmed that there are no amplification errors and that construction was accurate.

### Expression and purification of r-PilQ_380-706_

To construct an expression system, the coding sequence of *PilQ_380-706_*, whose theoretical molecular size is approximately 35 *kDa*, was constructed into expression vector pET28a to express a recombinant protein.

**Figure 2.**
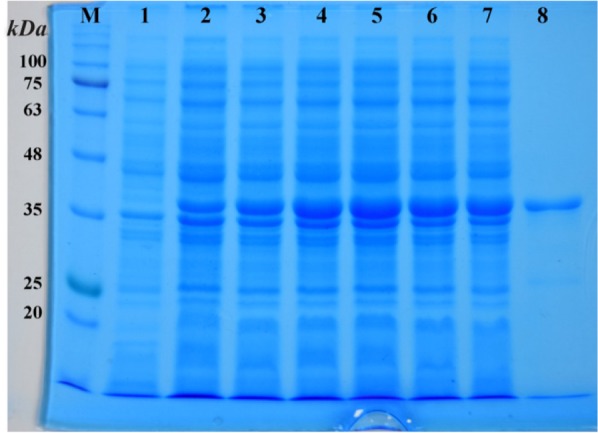
SDS-PAGE analysis of the expression of r-PilQ_380-706_ protein in *E. coli*. The total proteins of the BL21harboring pET28a/*pilQ*_1138-2118_ plasmid was harvested and loaded on 12% (*v/v*) SDS-PAGE after 4 *hr* induction with or without IPTG. (lane M) denote molecular weight marker proteins; (lanes 1) total cell lysate of non-induced bacteria; (lanes 2-7) 1-6 *hr* incubation after induction; (lane 8) purified r-PilQ_380-706_ after HisTrap Chelating and Ni^2+^-affinity chromatography (∼37 *kDa*).

**Figure 3.**
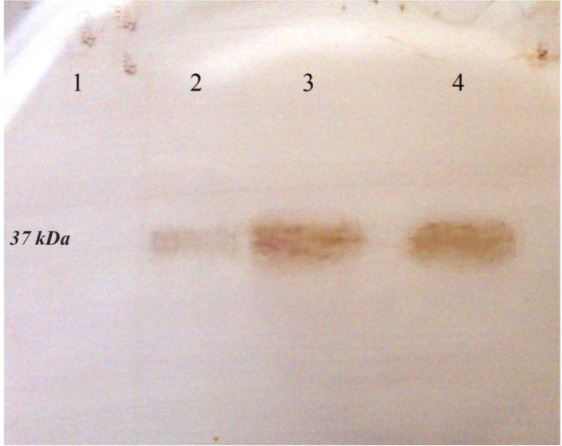
Western blot analysis of the expressed r-PilQ_380-706_ protein in *E. coli* BL21. After running the SDS-PAGE, the protein transferred onto PVDF membrane and detected with HRP-conjugated goat anti-rabbit IgG. (lane 1) total cell lysate of non-induced bacteria; (lane 2) total cell lysate of bacteria after 4 *hr* induction; (lane 3 and 4) purified r-PilQ_380-706_ by Ni^2+^-affinity chromatography.

The recombinant plasmid, pET28a*/pilQ*_1138-2118_, was transformed into *E. coli* BL21 (DE3). The His-Tagged recombinant protein was purified from inclusion bodies by using Ni^2+^-affinity chromatography. Results from SDS-PAGE analysis of expression products showed that the PilQ_380-706_ protein expressed 4 *hr* after induction with IPTG. The expression product of the protein was approximately 37 *kDa* in molecular size (Figure 2). The purified protein was confirmed by western blotting as showed in figure 3. The yield of the purified PilQ_380-706_ protein was about 2.18 *mg* per liter of culture media. In term of measured protein, no significant difference was observed between NanoDrop and Bradford protein assay.

### Production of PilQ_380-706_ -specific IgG and specificity analysis

**Figure 4.**
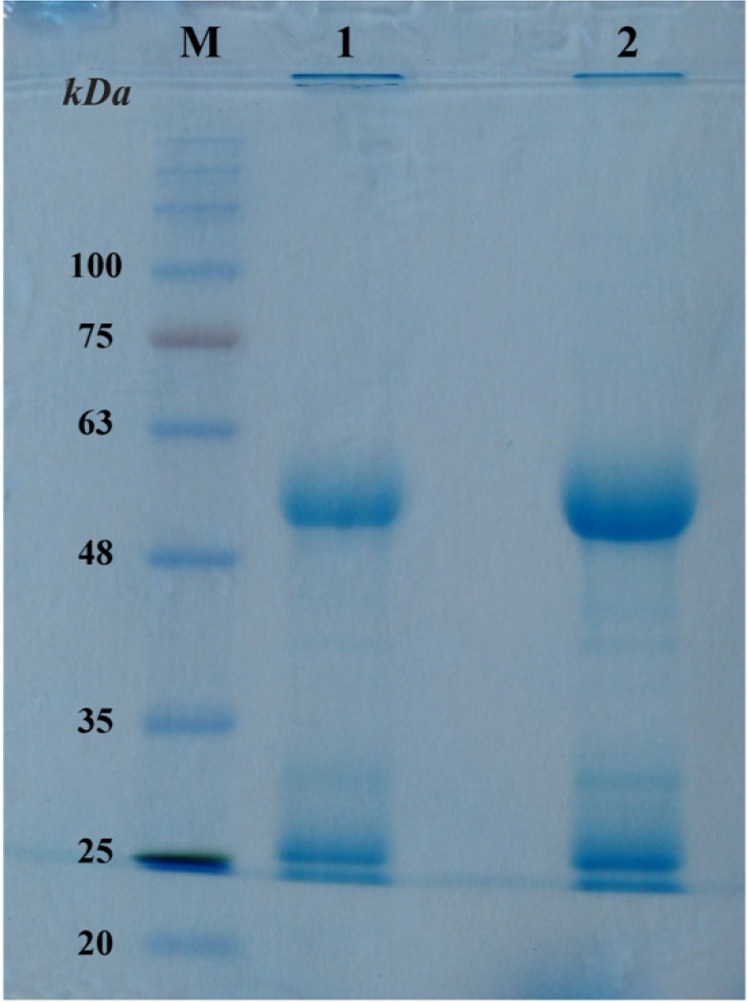
Coomassie blue-stained SDS-PAGE gel of affinity-purified antibodies. (lane M) molecular mass markers, in *kDa*; (lane 1) 10 *mM* glycine eluant; (lane 2) flowthrough from 100 *mM* glycine. Light chain (LC) and Heavy chain (HC) polypeptides were predicted to be 24.9 and 51.4 *kDa*, respectively.

Polyclonal antibodies against r-PilQ_380-706_ were produced in rabbit and finally the specific IgG was purified by using protein A/G agarose (Invitrogen, USA) according to manufacturer’s instruction. As shown in figure 4, β-Mercaptoethanol as a reducing agent break the hinge-region disulfide bonds and thus antibodies will dissociate into the heavy (51.4 *kDa*) and light (24.9 *kDa*) chains, respectively. To determine the specificity of the antiserum raised against purified r-PilQ_380-706_, western blot analysis was performed. The total cell extracts (induced and non-induced) and the purified PilQ_380-706_ protein were immunoblotted and then hybridized with specific polyclonal IgG. Addition of HRP-conjugated goat anti-rabbit IgG showed that the PilQ_380-706_ protein was substantially expressed by using the pET28a/*pilQ*_1138-2118_ expression vector when IPTG was added at the early-exponential phase of growth and collecting the cells 4 *hr* after induction (Figure 3). Overall, our results indicated that the rabbit produced antibodies are highly specific to detect the r-PilQ_380-706_.

### Twitching inhibition assay

**Figure 5.**
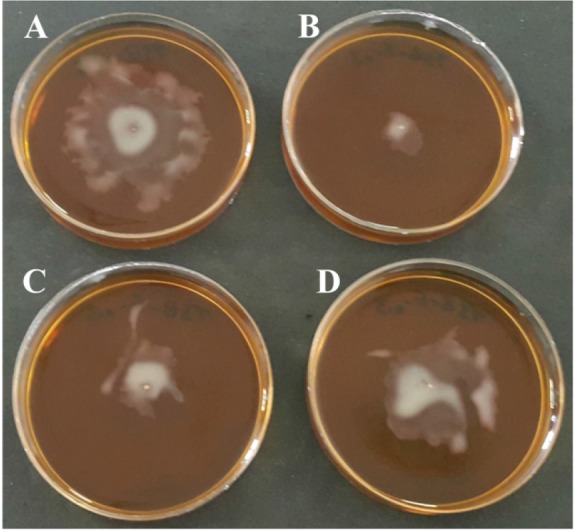
Twitching inhibition of *P. aeruginosa* PA01 strain in the presence of r-PilQ_380-706_ IgG on motility agar (LB broth with 1% *w/v* agar). The motility agar plates supplemented with either different concentrations of anti r-PilQ_380-706_ IgG or non-immune rabbit serum (NRS) were stab inoculated with *P. aeruginosa* and incubated for 18 *hr* at 37°*C*. Different concentrations (0.3, 0.2 and 0.1 *μg*) of antiserum raised against r-PilQ_380-706_ inhibited motility of *P. aeruginosa* PAO1 (b to d, respectively) compared with NRS (a).

Immunized and non-immunized rabbit sera were evaluated in the twitching inhibition assay for their biofunctional activity to inhibit the motility of PAO1 strain of *P. aeruginosa*. In this assay, NRS was used as control group. As shown in figure 5 and table 1, the rPilQ_380-706_ IgG was able to inhibit the motility of the PAO1 strain, so that the motility zone was significantly decreased compared to control group. In the presence of NRS, no immobilization was observed.

## Discussion

Among the various recombinant immunodominant antigens identified as a candidate vaccine against *P. aeruginosa*, the outer membrane proteins have shown promising potential. Hence, we tried to improve the purification conditions to obtain the recombinant protein in the pET28a vector system. In the present study, the secretin domain of the PilQ gene (*pilQ*_1138-2118_) from *P. aeruginosa* was designed into pET28a vector. This vector is one series of vectors without signaling regions allows accumulation of the PilQ_380-706_ protein in inclusion bodies (insoluble form). These types of proteins have been efficiently de- and renaturated. In addition, two 6×His-tag was designed at the N-and C-terminus of the r-PilQ_380-706_ coding sequence. His-tag, a small purification partner, has been designed into pET28a vector to decrease the time and cost of protein purification procedures without affecting protein well folding and bioactivity. The pET system was chosen because it is a very powerful system developed specially for the cloning, expression and purification of recombinant proteins in *E. coli* and also has been utilized to overexpress exogenous proteins for decades. The PilQ_380-706_ was expressed in BL21(DE3), carrying an inducible chromosomal gene for T7 RNA polymerase, which is controlled by an IPTG-inducible *lac*UV5 promoter. pET-series vectors also contain a *lac*I gene that provides *lac* repressor molecules to downregulate both the *lac*UV5-controlled chromosomal T7 RNA polymerase and the T7*lac* promoter ^18^. In the previous study, Fakhri *et al* cloned and expressed the conserved C-terminal fragment of the PilQ protein (PilQ_406-770_) from *Neisseria meningitidis* into pET28a vector. They demonstrated that, when the recombinant vector transform into prokaryotic expression system, high level of protein is produced following nickel affinity chromatography ^19^.

Most of the recombinant proteins thought aggregate as inclusion body, but can be solubilized and purified using hybrid condition. The significant increase in the yield of protein extraction from the inclusion bodies can be achieved by the addition of guanidine hydrochloride (G-HCl) as a solubilization agent. In this condition, the solubilized recombinant protein bind and wash under denature condition, and wash and elute under native conditions. Use of 6 M G- HCl alone as strong denaturant was sufficient for the solubilization of protein from inclusion bodies. In this study, we made the use of optimum concentration of non-ionic detergent (Triton X-100) that help to efficiently purify the r-PilQ_380-706_ from inclusion bodies. The use of 1% Triton X-100 helps to improve the lysis conditions of cells for further solubilization of inclusion bodies. Furthermore, after three rounds of washes under native conditions, the proportions of unrelated proteins were considerably decreased (data not shown). In addition, 5% (*v/v*) glycerol was added in the refolding buffer as it enhanced the stability and efficiently elevating the yield of protein.

In recent years, high-throughput protein-refolding techniques have been developed for renaturation of inclusion bodies ^20,21^. These include three methods such as dilution, dialysis or solid-phase separation for renaturation of inclusion bodies ^22^. In the present study, for improvement of the refolding process, we have selected dilution and dialysis methods. In on-column purification, we used a decreased gradient of urea for the gradual removal of urea and renaturation of recombinant protein. This washing process followed by dialysis (with buffer exchange), in which there was no protein precipitation and aggregation. Our efforts at refolding of the solubilized proteins using dilution and dialysis methods were led to effectively refolded desired recombinant protein. We found that after chromatography by Ni-NTA agarose, unrelated proteins further decreased, this was lead to an increase in the refolding yield and purity. In the present study, we not only evaluated the efficiency of pET28a vector for the expression of the r-PilQ_380-706_ but also simultaneously developed a highly reproducible and efficient procedure for purification and scalable production of the recombinant protein with high purity. The procedure developed here may be useful in the efficient purification of other recombinant proteins highly expressed in *E. coli* as inclusion bodies. Generally, the protein (PilQ_380-706_) tends to be expressed as inclusion bodies at lower temperature, but the rate of expression is more slowly for correct folding. Now that culture under lower temperatures are beneficial to stabilization of structure and expression of soluble protein, and the increase of temperature did not significantly enhance PilQ_380-706_ expression (data not shown), the following induction was carried out at 22°*C*. Overall, after several attempts to determine the optimal conditions, the highest amount of PilQ_380-706_ was produced by induction with 1 *mM* IPTG at 22°*C* for 4 *hr*.

The immunoreactivity of purified r-PilQ_380-706_ under modified conditions was examined *in vitro* by twitching inhibition assay. The twitching inhibition assay results show that the antiserum raised against the r-PilQ_380-706_ can inhibit cell motility of *P. aeruginosa* PAO1 *in vitro*. Immunoblot analysis demonstrated that the r-PilQ_380-706_-specific polyclonal IgG could detect the recombinant protein expressed in a prokaryotic cell (*E. coli* BL21). These findings indicate that the r-PilQ_380-706_ preserved correct folding. Since motility has been exhibited to be an important virulence factor in microbial pathogenesis ^23^, therefore, disruption of such a function by neutralizing and immobilizing antibodies *in vivo* may prove to be an advantageous prophylactic measure against pathogenic bacteria. These tests confirmed the bioactivity of the purified recombinant protein. Thus, the use of these reagents in the modified protocol does not have any adverse affects on the bioactivity of the protein. In the recent study, Koo *et al* showed that the absence of twitching motility of *P. aeruginosa* is correlated with the lack of PilQ multimer ^24^. We believe that, this is the first report on the expression and purification of the secretin domain of the PilQ_380-706_ protein with a His-tag in bacterial expression system. It is suggested that the r-PilQ_380-706_ could contribute as a vaccine or an adjuvant to control *P. aeruginosa* infection.

## Conclusion

In conclusion, the present study described a modified method for expression, purification and refolding of r-PilQ_380-706_ from *P. aeruginosa* in *E. coli*. The recombinant protein was expressed in the form of inclusion bodies under the pET28a expression vector. Here, we developed a reproducible and simplified method to achieve significant yields of the protein. The purification of r-PilQ_380-706_ was done under the modified hybrid condition. The procedure developed in this study may be useful in the efficient purification of other recombinant proteins expressed in *E. coli* as inclusion bodies. This recombinant protein was biologically active and recommended to be used as a vaccine or an adjuvant.
